# Intelligent Land-Vehicle Model Transfer Trajectory Planning Method Based on Deep Reinforcement Learning

**DOI:** 10.3390/s18092905

**Published:** 2018-09-01

**Authors:** Lingli Yu, Xuanya Shao, Yadong Wei, Kaijun Zhou

**Affiliations:** 1 School of Information Science and Engineering, Central South University, Changsha 410083, China; llyu@csu.edu.cn (L.Y.); 13477011934@163.com (Y.W.); 2 State Key Laboratory of Robotics and System, Harbin Institute of Technology, Haerbin 150001, China; 3 State Key Laboratory of Mechanical Transmissions, Chongqing University, Chongqing 400044, China; 4 School of Computer and Information Engineering, Hunan University of Commerce, Changsha 410205, China; alpha218@126.com

**Keywords:** intelligent driving vehicle, trajectory planning, end-to-end, deep reinforcement learning, model transfer

## Abstract

To address the problem of model error and tracking dependence in the process of intelligent vehicle motion planning, an intelligent vehicle model transfer trajectory planning method based on deep reinforcement learning is proposed, which is able to obtain an effective control action sequence directly. Firstly, an abstract model of the real environment is extracted. On this basis, a deep deterministic policy gradient (DDPG) and a vehicle dynamic model are adopted to jointly train a reinforcement learning model, and to decide the optimal intelligent driving maneuver. Secondly, the actual scene is transferred to an equivalent virtual abstract scene using a transfer model. Furthermore, the control action and trajectory sequences are calculated according to the trained deep reinforcement learning model. Thirdly, the optimal trajectory sequence is selected according to an evaluation function in the real environment. Finally, the results demonstrate that the proposed method can deal with the problem of intelligent vehicle trajectory planning for continuous input and continuous output. The model transfer method improves the model’s generalization performance. Compared with traditional trajectory planning, the proposed method outputs continuous rotation-angle control sequences. Moreover, the lateral control errors are also reduced.

## 1. Introduction

Although intelligent driving technology is developing rapidly, some new problems are emerging during development. In 2016, the first major accident of Tesla happened in the field of automatic driving. Meanwhile, Uber suffered an incident of automation driving hitting pedestrians on 28 March 2018. These problems greatly aroused worldwide attention on the safety of intelligent driving. Therefore, there is still a long way for intelligent driving to improve its innovative and stable safety. As the key to its technology, trajectory planning technology is attracting more and more attention and exploration by researchers at home and abroad.

Trajectory planning is not only applied to intelligent vehicles, but also widely used in the field of robotics and unmanned aerial vehicles [1,2]. There are various ways of trajectory generation in trajectory planning, including the Nelson polynomial, spiral curve equation, spline curve, Bezier curve, etc. [3]. For example, a fourth-order polynomial and dynamic bicycle model were utilized to describe a vehicles kinematics model [4], considering the overtaking and chasing behavior of different cost functions in each case. However, it assumes that the vehicle velocity is a constant, which conflicts with most actual situations. Yu, L et al. [5] put forward a technique of trajectory smoothing and stitching based on a Bezier Curve. Sahingoz, O.K. [6] proposed trajectory planning based on a Bezier Curve that takes into consideration the kinematics constraint, initial state constraint, target state constraint, and curvature continuous constraint.

With the rapid development of deep learning, vision-based control methods acquired great achievements [7]. Hotz [8] adopted a variational auto-encoder (VAE) and a generative adversarial network (GAN) to achieve image coding, road tracking, and intelligent driving vehicle potential space decoding. Low-level control strategy and advanced prior action were learned through a neural network, and multi-level strategies were taken as heuristic search algorithms to realize complex motion planning tasks [9]. Deep learning models were adopted to establish the mapping relationship between lidar distance, target position, and control instruction [10]. To realize the motion planning of an intelligent vehicle, Liu, W et al. [11] and Lin, Y.L et al. [12] proposed deep learning to establish the mapping relationship between the control sequence and the corresponding trajectory.

In recent years, reinforcement learning was applied to robot control tasks. A deep Q network (DQN) was proposed to deal with a discrete action continuous state, which aimed to combine a deep neural network with reinforcement learning [13]. Subsequently, Lillicrap, T.P et al. [14] and Gu, S et al. [15] offered an offline depth reinforcement learning algorithm based on a deep Q network and extended it to continuous high-dimensional state space. Schaul, T et al. [16] and Metz, L et al. [17] made it possible to achieve multiple targets by extending the DQN. Schaul, T et al. [18] proposed prior experience replay technology to improve the performance of the DQN. Andrychowicz, M et al. [19] exposited experience to improve sample collection efficiency. The Ornstein–Uhlenbeck (OU) process [20] was used to add noise after an action strategy to improve network exploration ability. Plappert, M et al. [21] introduced network parameter hierarchy with noise to improve network performance. Gu, S et al. [22] showed the possibility of learning complex manipulation strategies without demonstrations. Genders, W et al. [23] adopted a deep reinforcement learning model to establish a traffic signal agent, while Isele, D et al. [24] solved the problem of a complex traffic intersection without traffic signals. Tai, L et al. [25] proposed a motion planning method without a map. The sparse sensor ranging information and target position were utilized as input, while the continuous steering command was taken as output, and verification was conducted in practical experiments. Data efficiency and task performance were improved by addressing the problem of maximizing cumulative rewards for reinforcement learning (RL), and considering supervised/unsupervised learning styles, so as to achieve navigational capabilities [26].

When the model is known, a strategy iteration and value iterative algorithm based on dynamic programming (DP) [27] is able to update value functions after each step of the strategy, which is efficient. The intelligent vehicle driving problem is a model-free problem. The Monte Carlo (MC) [28] reinforcement learning algorithm overcomes the difficulties caused by the unknown model estimation by considering the sampling trajectories. This algorithm updates the value estimate of the strategy after completing a sampling trajectory, which is inefficient compared to the algorithm based on dynamic programming. Temporal difference (TD) learning [29] combines dynamic programming and Monte Carlo reinforcement learning for more efficient model-free learning.

As known, Q-learning solves the low-dimensional problem of discrete space, which is a classic case of temporal difference learning. The DQN improves the processing ability of high-dimensional state space, but it is still unable to cope with high-dimensional continuous action space. The Actor-Critic (AC) method [30] is able to handle continuous action space, but the randomness strategy makes it difficult for the network to converge. To this end, the deep deterministic policy gradient (DDPG) [31] adopts the Actor-Critic framework to combine the advantages of DQN to solve the problem of continuous state space and continuous action space. Moreover, it adopts a deterministic policy to ensure the network is more convergent. Since traditional motion cannot eliminate the model error, an end-to-end model transfer trajectory planning method based on depth reinforcement learning is proposed in this study. Furthermore, DDPG is a deep reinforcement learning method, and it is utilized to train the model in a simple virtual environment which is constructed independently, thereby reducing its dependence on the sample data. Additionally, it can deal with the model training of continuous input and continuous output, thus directly outputting the control action and trajectory sequence. The complexity is lower than that of the optimal control calculation, and the model transfer method was applied to improve the model’s generalization performance. Compared with traditional planning and end-to-end planning, the proposed method has more continuous corner control sequences and smaller lateral errors while the vehicle is driving.

## 2. Reinforcement Learning and Description of the Driving Environment

### 2.1. Reinforcement Learning Method

**Figure 1:**
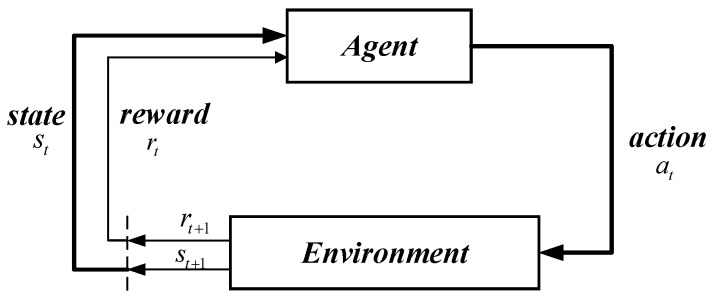
The principle of reinforcement learning.

The basic principle of reinforcement learning is presented in Figure 1. When the agent is required to achieve a task, it first interacts with environment (***Env***) via action (***a***); then, the impact of the action on the environment brings the agent into a new state (***s***). At the same time, the agent receives reward feedback (***Reward***) from the environment. The agent and environment generate a large amount of data through a continuous loop and interaction. Reinforcement learning utilizes these sample data to adjust the strategy ***π***. Afterward, it interacts with ***Env*** to enter a new ***state***, generating new $data=(s_{t},a_{t},r_{t},s_{t+1})$. Subsequently, the new samples are adopted to modify the strategy ***π*** for several iterations. After a great deal of iterative learning, the agent finally learns the optimal strategy π* to complete the corresponding task.

Strategy ***π*** refers to the agent’s selection of action ***a*** under state ***s***, which is the key problem in reinforcement learning. Strategy ***π*** is a map from the agent, which is aware of environmental state ***s*** to action ***a***. The random strategy selects the corresponding action according to the probability π(a|s) of each action, while deterministic policy selects action a=π(s) directly according to ***s***.

(1) $$\begin{array}{l} stochastic\;Policy:\;\sum \pi (a|s)=1\\ deterministic\;Policy:\;\pi (s):S\to A \end{array}.$$

Cumulative rewards or returns are calculated when a strategy ***π*** is given. The definition of cumulative returns is as follows:

(2) $$G_{t}=R_{t+1}+\gamma R_{t+2}+\cdots =\sum_{k=0}^{\infty }\gamma^{k}R_{t+k+1},$$

where 0<γ<1 is the discount factor of long-term income. The state function is defined as the cumulative return benefit corresponding to state ***s*** under ***a*** strategy ***π***:

(3) $$\upsilon_{\pi }(s)=E_{\pi }\left[\sum_{k=0}^{\infty }\gamma^{k}R_{t+k+1}|S_{t}=s\right].$$

The corresponding state-action value function is defined as

(4) $$q_{\pi }(s,a)=E_{\pi }\left[\sum_{k=0}^{\infty }\gamma^{k}R_{t+k+1}|S_{t}=s,A_{t}=a\right].$$

### 2.2. Virtual Driving Environment Model Design

**Figure 2:**
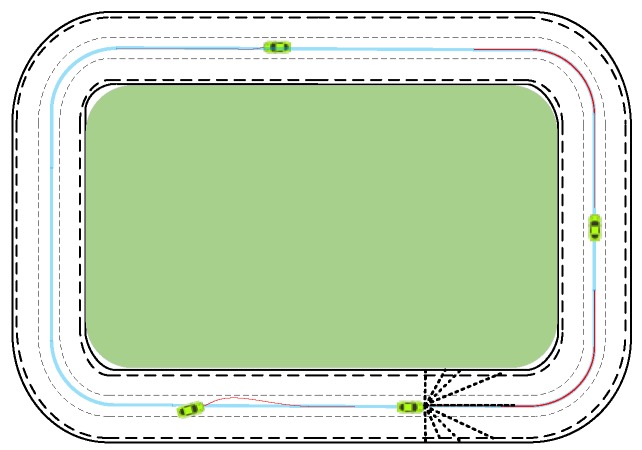
Virtual environment abstract model.

According to the description of the intelligent vehicle driving scene, intelligent driving behavior decision tasks include normal driving, changing/overtaking, curve/ramp driving, and so on. Here, the environment model was built as a circular map with three lanes, as shown in Figure 2. Specifically, the green area and outer lane boundary are deemed insurmountable obstacles, while the others are free travel space. The light-blue lines indicate the desired path with rewards, $path_{d}=\left(X_{d},Y_{d},\phi_{d}\right)$. The intelligent vehicle, $Car=(x_{c},y_{c},\varphi_{c},v)$, drives in a circular map and learns intelligent driving maneuvers, including straight, changing, and curving driving behavior. Lastly, $x_{c},y_{c},\varphi_{c}$ represents the current position and posture information of the intelligent vehicle.

To simplify the environment model ***Env,*** the intelligent vehicle has ***n*** ranging beams ***Sensor***. Furthermore, the farthest distance $d_{Max}$ of each ranging beam is the same. Each ranging beam supports feedback information $Sensor_{i}=(d_{i},x_{end},y_{end})$ to the intelligent vehicle when it encounters obstacles. Here, $d_{i}$ is the length that the ranging beam is blocked by obstacles or boundaries, and $x_{end},y_{end}$ are the position coordinates of the beam in contact with obstacles or boundaries. The intelligent vehicle speed keeps a constant v, and the angle control output is $-\delta_{\min}\leq \delta_{t}\leq \delta_{\max}$. Thus, the key return function ***Reward*** for the environment model ***Env*** is as follows:

(5) $$Reward=\left\{ \begin{array}{ll} -1 & when\;contacts\;with\;obstacles\;or\;boundaries\\ R_{action}+R_{money} & else \end{array}\right.,$$

(6) $$\{\begin{array}{c} R_{action}=-\lambda_{1}\times {\left\|\delta_{old}-\delta \right\|}^{2}\\ R_{money}=\left\{ \begin{array}{ll} 0 & get\_money\;=\;False\\ 0.1 & get\_money=True \end{array}\right. \end{array},$$

(7) $$get\_money\;=\;\left\{ \begin{array}{ll} True & if\;\left|\Delta x\right|\leq \varepsilon_{1}\;\&\;\left|\Delta y\right|\leq \varepsilon_{2}\;\&\;\left|\Delta \varphi \right|\leq \varepsilon_{3}\\ False & other\;else \end{array}\right.,$$

where $\lambda_{1}$ is the positive penalty coefficient, and $R_{action}$ represents the difference penalty between the front and rear successive front wheel angles $\delta_{old},\;\delta$ of an intelligent vehicle. The smaller the change is between successive actions, the smaller the penalty. $R_{money}$ represents the reward for an intelligent vehicle driving on the desired path, (Δx,Δy,Δφ) is the difference between the current posture $\left(x_{c},y_{c},\varphi_{c}\right)$ and the desired path $path_{d}=\left(X_{d},Y_{d},\phi_{d}\right)$, and $\varepsilon_{1},\varepsilon_{2},\varepsilon_{3}$ is the fault-tolerant error.

Intelligent vehicles randomize their initial position $\left(x_{0},y_{0},\varphi_{0}\right)$ according to the given policies $\pi_{reset}$ to ensure a more adequate exploration of the environment and the stability of the result. Intelligent vehicle termination conditions at each epoch include 1) contact with obstacles or boundaries; 2) meeting the maximum number of driving steps, $n_{step}=Num_{\max}$. The optimal intelligent driving maneuver for intelligent vehicle learning is π; therefore, the strategy space of the model is $\pi_{all}=\left\{\pi_{reset},\pi \right\}$.

(8) $$\pi_{reset}:\left\{ \begin{array}{l} x_{0}\in [X_{\min},X_{\max}]\\ y_{0}\in [Y_{\min},Y_{\max}]\\ \varphi_{0}\in [\phi_{\min},\phi_{\max}] \end{array}\right..$$

State space is assumed as $\sum (Sensor)=\left\{d_{0},d_{1},\ldots ,d_{n}\right\}$, and motion space is assumed as $\sum (\delta )=\left\{\delta_{new}\right\}$. For the intelligent vehicle and environment model ***Env***, an abstract model ***M*** is constructed.

$$M=\left\{Env,Car,\sum (Sensor),\sum (\delta ),\pi_{all},Reward\right\}.$$

## 3. Model Transfer Trajectory Planning Based on Deep Reinforcement Learning (DRL-MTTP)

### 3.1. DDPG Network Structure and Algorithm Flow

**Figure 3:**
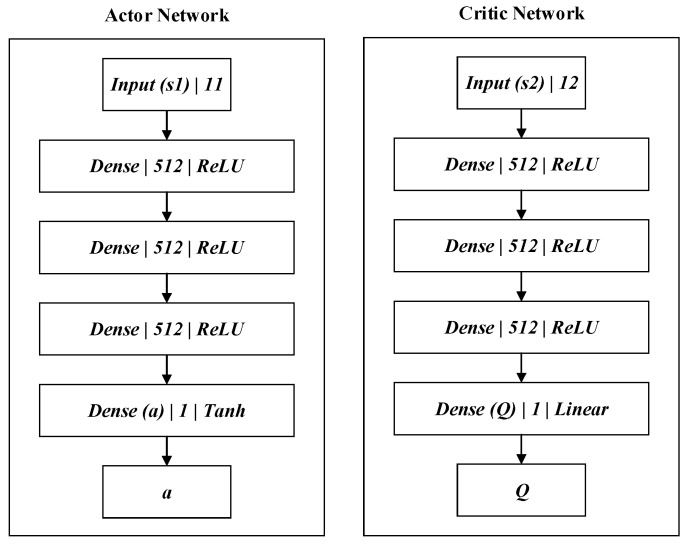
Deep deterministic policy gradient (DDPG) network structure.

For complex continuous state space ∑(Sensor) and continuous action space ∑(δ), it is necessary to train the deep reinforcement learning model $M_{\theta }$ in a virtual environment ***M*** using the DDPG algorithm. The DDPG algorithm consists of an Actor policy network and a Critic evaluation network, as shown in Figure 3. 

The state $s_{sensor}\in \sum (Sensor)$, speed v, and action $\delta_{old}$ of the final moment are combined as $s_{a}=\left(s_{sensor},v,\delta_{old}\right)$. They are adopted as the input to the Actor policy network; therefore, the number of Actor policy network input-layer neurons is 11. Meanwhile, the policy network’s hidden layer utilizes three full connected networks; each layer contains 512 neurons. The fully connected layer is followed by batch normalization (BN), before the *ReLU* (a type of activation function) is adopted as the activation function. At the same time, the last layer of the network chooses *tanh* as the activation function to map the network output between the interval [−1, 1]. The network output is action δ∈∑(δ). After the state $s_{a}$ and action δ are merged as $s_{c}=(s_{a},\delta )$, then they become the input to the Critic evaluation network. The number of Critic policy network input-layer neurons is 12. Meanwhile, the policy network’s hidden layer utilizes three full connected networks; each layer contains 512 neurons. The fully connected layer is followed by BN. Although the hidden layer of the evaluation network and the policy network hold the same structure, its last layer is activated by a linear function such as that in Equation (9). Thus, the network output is the corresponding Q-value, $Q(s_{a},\delta )$, of $s_{c}$.

(9) y=kx+b,

where x is the input of the last layer, y is the predicted Q-value, and k,b are the weight and bias for network training.

**Figure 4:**
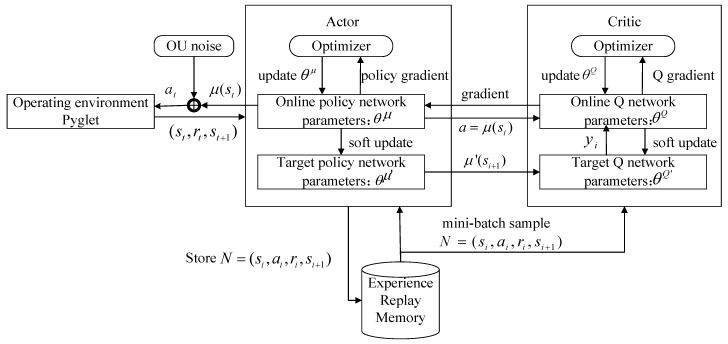
DDPG algorithm flow chart. OU—Ornstein–Uhlenbeck process.

DDPG adopts the Actor-Critic framework, including the Actor and Critic structure. Here, the Actor part includes the online policy network and the target policy network, which adopt the deterministic policy to get a definite action from the current state. The Critic part includes an online Q network and a target Q network, in which the Bellman equation of the action–state function Q is utilized to measure the quality of action. The pseudo code of the DDPG algorithm is shown in Algorithm 1 and the DDPG algorithm flow is shown in Figure 4. The input state $\left(d_{1},\ldots ,d_{9},v,\delta_{old}\right)$ and output action δ are represented accordingly. The DDPG algorithm adopts a deterministic policy, and the policy output is an action. Therefore, it needs less sampled data to maximize efficiency. However, this results in the environment not being explored. In order to improve the algorithm’s exploration ability, the OU stochastic process was added to the deterministic policy action. Furthermore, the environmental execution was carried out after sampling from a random process of the action. Due to its good correlation over a time series, the OU stochastic process is able to explore environments with momentum properties by the agent.


**Algorithm 1. Pseudo code of the deep deterministic policy gradient (DDPG) algorithm. OU—Ornstein–Uhlenbeck process.**

$\mathrm{Randomly}\;\mathrm{initialize}\;\mathrm{Critic}\;\mathrm{online}\;Q\;\mathrm{network}\;\mathrm{parameters}\;\theta^{Q}$$\;\mathrm{and}\;\mathrm{Actor}’s\;\mathrm{online}\;\mathrm{policy}\;\mathrm{network}\;\mathrm{parameters}\;\theta^{\mu }$.$\mathrm{Initialize}\;\mathrm{Critic}\;\mathrm{target}\;Q\;\mathrm{network}\;\mathrm{parameters}\;\theta^{Q^{\prime }}\leftarrow \theta^{Q}$$\;\mathrm{and}\;\mathrm{Actor}’s\;\mathrm{target}\;\mathrm{policy}\;\mathrm{network}\;\mathrm{parameters}\;\theta^{\mu^{\prime }}\leftarrow \theta^{\mu }$.Initialize experience replay memory (R).for *episode* = 1, M do Initialize the OU random process *D* for the exploration of action. $\mathrm{Input}\;\mathrm{initial}\;\mathrm{observation}\;\mathrm{state}\;s_{1}$. for t=1, T do  $\mathrm{Choose}\;\mathrm{action}\;a_{t}$$\;\mathrm{based}\;\mathrm{on}\;\mathrm{current}\;\mathrm{strategy}\;\mu \left(s_{t}\right)$$\;\mathrm{and}\;\mathrm{exploring}\;\mathrm{noise}\;D_{t}$: $a_{t}=\mu \left(s_{t}\right)+D_{t}$  $\mathrm{Perform}\;\mathrm{the}\;\mathrm{action}\;a_{t}$$,\;\mathrm{get}\;\mathrm{the}\;\mathrm{reward}\;r_{t}$$,\;\mathrm{and}\;\mathrm{observe}\;\mathrm{the}\;\mathrm{new}\;\mathrm{state}\;s_{t+1}$.  $\mathrm{Store}\;\mathrm{the}\;\mathrm{process}\;\left(s_{t},a_{t},r_{t},s_{t+1}\right)$ in *R*.  $\mathrm{Sampling}\;\mathrm{from}\;R\;\mathrm{to}\;\mathrm{get}\;\mathrm{the}\;\mathrm{process}\;\left(s_{i},a_{i},r_{i},s_{i+1}\right)$ of batch *N*.  $\mathrm{Set}\;y_{i}=r_{i}+\gamma Q^{\prime }\left(s_{i+1},\mu^{\prime }\left(s_{i+1}|\theta^{\mu^{\prime }}\right)|\theta^{Q^{\prime }}\right)$$//\;Q^{\prime }$$\;\mathrm{is}\;\mathrm{the}\;\mathrm{state}\text{–}\mathrm{action}\;\mathrm{value}\;\mathrm{calculated}\;\mathrm{by}\;\mathrm{the}\;\mathrm{target}\;Q\;\mathrm{network},\;\mathrm{and}\;\mu^{\prime }$ is the current strategy obtained by the target policy network.  $\mathrm{Update}\;\mathrm{Critic}’s\;\mathrm{online}\;Q\;\mathrm{network}\;\mathrm{by}\;\mathrm{minimizing}\;\mathrm{the}\;\mathrm{loss}\;\mathrm{function}:\;L=\frac{1}{N}\sum_{i}\left(y_{i}-Q{\left(s_{i},a_{i}|\theta^{Q}\right)}^{2}\right)$  $\mathrm{Update}\;\mathrm{the}\;\mathrm{Actor}’s\;\mathrm{online}\;\mathrm{policy}\;\mathrm{network}\;\mathrm{with}\;\mathrm{sampling}\;\mathrm{gradient}:\;\nabla_{\theta^{\mu }}\mu |s_{i}\approx \frac{1}{N}\sum_{i}\nabla_{a}Q\left(s,a|\theta^{Q}\right){|}_{s=s_{i},a=\mu \left(s_{i}\right)}\nabla_{\theta^{\mu }}\mu \left(s|\theta^{\mu }\right){|}_{s_{i}}$  $\mathrm{Update}\;\mathrm{Critic}’s\;\mathrm{target}\;Q\;\mathrm{network}:\;\theta^{Q^{\prime }}\leftarrow \tau \theta^{Q}+\left(1-\tau \right)\theta^{Q^{\prime }}$  $\mathrm{Update}\;\mathrm{Actor}’s\;\mathrm{target}\;\mathrm{policy}\;\mathrm{network}:\;\theta^{\mu^{\prime }}\leftarrow \tau \theta^{\mu }+\left(1-\tau \right)\theta^{\mu^{\prime }}$ end forend for


### 3.2. Model Transfer Strategy

The intelligent vehicle ***Car*** obtains the optimal intelligent driving strategy π from the environment model ***M*** through deep reinforcement learning training. The real environment M* is obviously different from the virtual environment model ***M***; the former usually tends to be more complex and time-varying. Therefore, if the training model of virtual environment ***M*** is directly applied to real environment M*, it brings numerous predictable or unpredictable problems.

The decision tasks of intelligent vehicles include driving straight, changing lanes, crossing corners, ramp driving, etc. Thus, the model of intelligent driving tasks is abstracted from the real environment M* and migrated to the virtual environment ***M***, which maps onto a location area corresponding to the ring map in ***M***. Then, the optimal driving strategy π is adopted to plan the control-trajectory sequence C={δ,ζ} to achieve the driving task, including the control sequence $\delta =\left\{\delta_{1},\delta_{2},\ldots ,\delta_{t}\right\}$ and its corresponding trajectory $\zeta =\left\{p_{1},p_{2},\ldots ,p_{t}\right\}$. Finally, the intelligent vehicles carry out the task δ to complete the driving task in the real environment M*.

According to a different sub-task ℤ, such as lane keeping, lane changing, and overtaking, the fixed reference points $P_{ref}|\mathbb{Z}=\left(x_{ref},y_{ref},\varphi_{ref}\right)$ in ***M*** are set as a reference target or local tasks. In terms of the driving task’s endpoint goal $P_{tar}=\left(x_{tar},y_{tar},\varphi_{tar}\right)$ of path planning and intelligent vehicle posture $Car_{M*}=\left(x_{c|M*},y_{c|M*},\varphi_{c|M*}\right)$ in the real environment M*, the model transfer strategy ℜ is mapped to ***M*** using Equation (10). Finally, the intelligent parking posture is obtained as $Car=\left(x_{c},y_{c},\varphi_{c}\right)$.

(10) $$ℜ:\left\{ \begin{array}{l} \theta =\varphi_{tar}-\varphi_{ref}\\ \left(x^{\prime },y^{\prime },\varphi^{\prime }\right)=\left(x_{c|M*}\cos\theta -y_{c|M*}\sin\theta ,x_{c|M*}\sin\theta +y_{c|M*}\cos\theta ,\varphi_{c|M*}-\theta \right)\\ \left(x_{tar}{}^{\prime },y_{tar}{}^{\prime },\varphi_{tar}{}^{\prime }\right)=\left(x_{tar}\cos\theta -y_{tar}\sin\theta ,x_{tar}\sin\theta +y_{tar}\cos\theta ,\varphi_{ref}\right)\\ \left(\Delta x,\Delta y,\Delta \varphi \right)=\left(x^{\prime }-x_{tar}{}^{\prime },y^{\prime }-y_{tar}{}^{\prime },\theta \right)\\ \left(x_{c},y_{c},\varphi_{c}\right)=\left(x_{ref}+\Delta x,y_{ref}+\Delta y,\varphi_{ref}+\Delta \varphi \right) \end{array}\right.,$$

where θ is the difference between target heading angle $\varphi_{tar}$ of the vehicle’s driving destination and heading angle $\varphi_{tar}$ of the reference point. In order to keep the heading angle of the target point $P_{tar}$ in the real environment coinciding with the heading angle of reference point $P_{ref}$ in the virtual environment, the real environment coordinate system is rotated by $\left(x^{\prime },y^{\prime },\varphi^{\prime }\right)$, which is the pose corresponding to the ego vehicle in the rotated coordinate system, and $\left(x_{tar}{}^{\prime },y_{tar}{}^{\prime },\varphi_{tar}{}^{\prime }\right)$, which is the pose corresponding to the target point *P* in the rotated coordinate system. (Δx,Δy,Δφ) is the difference in pose between the ego vehicle and the target point in the rotated coordinate system, and $\left(x_{c},y_{c},\varphi_{c}\right)$ is the corresponding pose of the ego vehicle in the virtual environment after the model transfer. 

From Figure 2, the virtual environment seems to contain only two curves, but this is inaccurate. After the training is completed, the vehicle can not only complete straight and turning tasks, but also complete lane-changing operations. Trajectories generated in the lane-changing phase contain different curvatures; thus, mapping ramps to the lane-changing phase in stages can solve the problem of ramp driving. 

However, the virtual simulation environment does not consider turning around; thus, when the actual road curvature is very large, the proposed method is not applicable.

**Figure 5:**
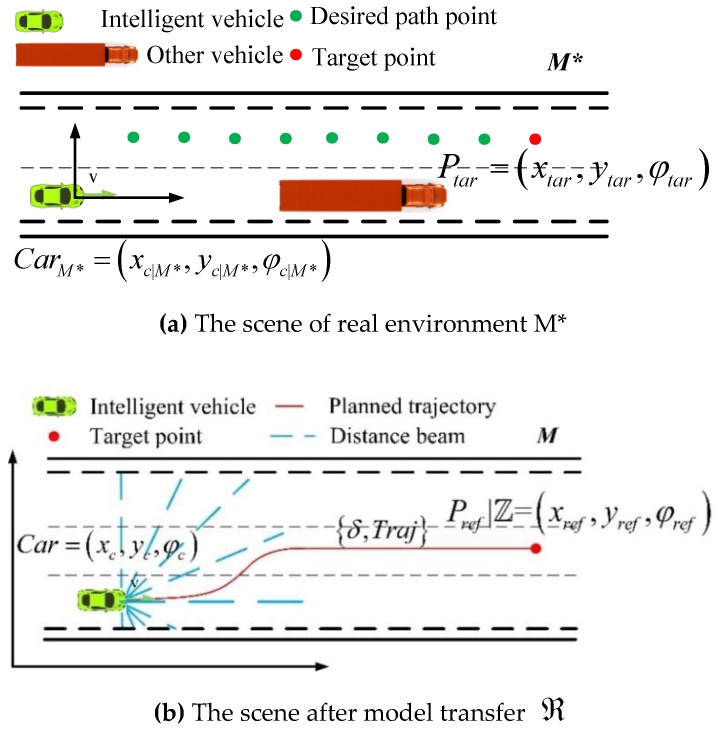

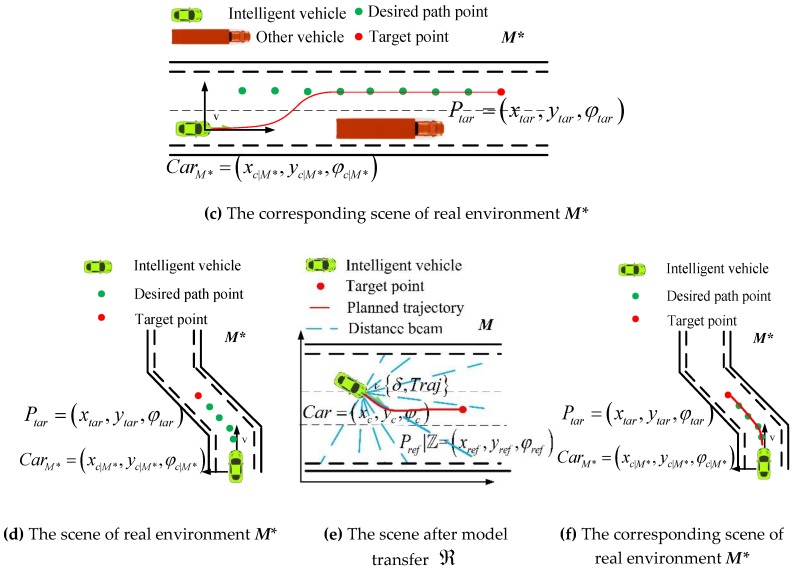
Transition diagram of the switch task model.

Figure 5a–c show the model transfer process of a lane change. Figure 5a shows the real-world M* scene. The road condition information is composed of invariants and variables. The invariants include the number of lanes and the width of lanes. The variables are the information regarding obstacles, which is fed back to the distance beam of the intelligent vehicle. The initial planning process is to travel along the current driving lane. When it is detected that there is a vehicle with lower speed than the ego vehicle ahead of it in the current lane, the left lane is taken as a desired path. The driving task is to switch to the left lane based on behavioral decision planning.

Figure 5d–f show the model transfer process of a ramp. Figure 5d shows the real-world M* scene.

The green vehicle is the current vehicle position, $x={\left[x,y,\varphi ,v,\omega \right]}^{T}$, and the pose information is $Car_{M*}=\left(x,y,\varphi \right)$. The green dot in the center of left lane is the scattered point of path planning. Here, the red point is the destination of current driving task $P_{tar}=\left(x_{tar},y_{tar},\varphi_{tar}\right)$. Figure 5b,e show the scene after $Car_{M*},P_{tar}$ is migrated to ℜ through the model. Mapping to the virtual environment ***M***, the intelligent vehicle pose is $Car=(x_{c},y_{c},\varphi_{c})$. ∑(Sensor) is acquired according to the distance beam in ***M***; then, $\delta_{old}$ is merged into state s, and the control sequence $\delta =\left\{\delta_{1},\delta_{2},\ldots ,\delta_{t}\right\}$ and trajectory sequence $\zeta =\left\{p_{1},p_{2},\ldots ,p_{t}\right\}$ are obtained by the model $M_{\theta }$. Figure 5c,f show the corresponding scene of control sequence $\delta =\left\{\delta_{1},\delta_{2},\ldots ,\delta_{t}\right\}$ and track sequence $\zeta =\left\{p_{1},p_{2},\ldots ,p_{t}\right\}$ in the real environment M*.

### 3.3. Algorithm Framework of Model Transfer Based on Deep Reinforcement Learning

**Figure 6:**
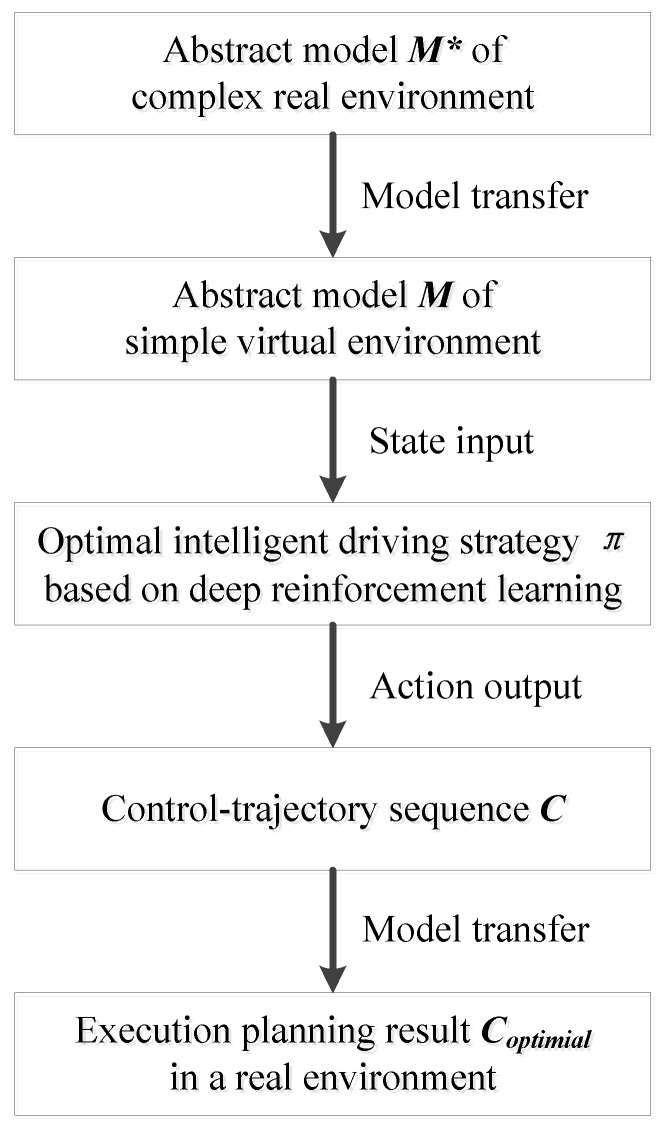
Block diagram of model transfer trajectory planning based on deep reinforcement learning (DRL-MTTP).

DRL-MTTP aims to abstract the complex real environment and transfer it to a simple virtual environment through the model. Furthermore, the optimal intelligent driving strategy is applied to the virtual environment, which is trained by the agent after deep reinforcement learning. Thus, the optimal trajectory control sequence is obtained to realize the end-to-end trajectory planning in the real environment. Figure 6 shows a technical diagram of DRL-MTTP.

The framework of DRL-MTTP is shown in Algorithm 2. Firstly, sub-task γ and path-planning datasets are initialized according to upper data streams. Afterward, the trajectory-planning stage begins. Then, an appropriate target point $P_{target}$ is selected as the local goal based on the sub-task γ. Thus, the selection process is shown in Equation (11).

(11) $$\left\{ \begin{array}{l} s_{t-1,t}=\sqrt{{\left(x_{t}-x_{t-1}\right)}^{2}+{\left(y_{t}-y_{t-1}\right)}^{2}}\\ l_{}{}^{2}=\sum_{t=1}^{target}s_{t-1,t} \end{array}\right.,$$

where $\left(x_{i},y_{i}\right)$ are the corresponding coordinates of $P_{i}$, $s_{t-1,t}$ is the straight-line distance between $P_{t-1}$ and $P_{t}$, l is the arc length threshold of path scatter points which is determined by sub-task γ, and $P_{target}$ is the target point that satisfies the threshold requirement. Subsequently, the target set is obtained by adding noise to the target point $P_{target}$, and $\left(\varepsilon_{x},\varepsilon_{y},\varepsilon_{\varphi }\right)$ satisfies a Gaussian distribution. Furthermore, the corresponding position Car of the intelligent vehicle in the virtual environment M is calculated by a model transfer for each target point $P_{target}$. The status of the environment ***s*** and the status of the intelligent vehicle ***x*** are gained by observing its ranging light beam in M.

During the planning time T, the deep reinforcement learning model $M_{\theta }$ is utilized in each unit of time ***t*** to analyze state s and predict the action $\delta_{t}$. At the same time, the dynamic model is adopted to simulate the prediction action. Meanwhile, the environment state s and intelligent vehicle state *x* are updated, and the track $\zeta_{t}$ is recorded. Finally, the control-trajectory sequence pair C={δ,ζ} under real environment M* is acquired by model transfer.

The second stage is about optimal trajectory selection. In this stage, the evaluation function of each control-trajectory pair is calculated. At the same time, the collision probability of the trajectory ζ is also judged. The control-trajectory pair with minimum ***J*** and no collision trajectory are taken as the optimal trajectory.

(12) $$\min J=\kappa_{1}\int_{0}^{T}\left(\Delta \delta^{2}\right)dt+\kappa_{2}\left[h\left(\zeta_{T}\right)-h\left(\zeta_{target}\right)\right],$$

where ***T*** is the termination time, Δδ is the difference of continuous action, $\kappa_{1},\kappa_{2}$ is the weight coefficient, and h represents the rectangular area of the vehicle outline at the end of its trajectory.

Finally, the result of planning is executed. The intelligent vehicle implements the first τ steps of control sequence δ, and the model reference control is utilized to enhance the robustness and to reduce the influence of system error by the model error. If the task is not terminated, planning action of the first two phases is repeated to realize the intelligent vehicle dynamic trajectory planning.


**Algorithm 2. Model transfer trajectory planning based on deep reinforcement learning (DRL-MTTP) frame.**

initialize ***terminal***, $S_{1}:\left\{P_{1},P_{2},\ldots ,P_{m}\right\}$; **//**receive tasks and data from the top
**while** terminal=false
 **//**trajectory planning stage $P_{target}\overset{}{\leftarrow }S_{1}:\left\{P_{1},P_{2},\ldots ,P_{m}\right\}$; **//s**elect a target from a path planning scatter set $S_{1}$ based on a task **for** i=0, N **do**  $P_{target,i}=P_{target}+\xi_{noise}$; //add noise to generate target sets  $Car_{i}\overset{ℜ}{\leftarrow }\left(P_{target},P_{ref},Car_{M*}\right)$; **//**calculate the corresponding position and pose of the intelligent vehicle by model transfer  $s,x\overset{M}{\leftarrow }\left(Car_{i},Sensor\right)$; **//**calculate state based on ranging light beam  **for** t=0, T **do**   $\delta_{t}\overset{M_{\theta }}{\leftarrow }s$; **//**calculate action by deep reinforcement learning model   $x=\int_{0}^{\Delta t}f\left(x,\delta_{i}\right)$; **//**dynamic model simulation   $\zeta_{i}\leftarrow x$; **//**record track  **end for**  $C_{i}:\left\{\delta ,\zeta *\right\}\overset{ℜ}{\leftarrow }\left(\delta ,Car_{i},Car_{M*},\zeta \right)$; **//**calculate control-trajectory sequence pair by model transfer **end for** **//**optimal trajectory selection stage $J_{\min}=\infty$; **for** i=0, N **do**  $J=\kappa_{1}\int_{0}^{T}\left(\Delta \delta^{2}\right)dt+\kappa_{2}\left[h\left(\zeta_{T}\right)-h\left(\zeta_{target}\right)\right]$; **//**calculate and obtain evaluation function  **if** $J<J_{\min}$ **and** no collision //select the control-trajectory pair with minimum *J* value and no collision as the optimal trajectory   $J_{\min}=J$;   $C_{optimal}=C_{i}$;  **end if** **end for** //stage of execute the planning result $\delta_{optimal}:\left\{\delta_{1},\delta_{2},,,,\delta_{\tau }\right\},\tau \leq T$; **//**get the results of the first τ steps update ***terminal***; **//**whether the task ends or not
**end while**



## 4. Simulation Test on Trajectory Planning of Intelligent Vehicle

### 4.1. Deep Reinforcement Learning Model Training

In the virtual simulation environment ***Env***, the circle map was 100 m long and 50 m wide. The driveway was 3.4 m wide. Here, the speed was v=36 km/h, which was kept constant. The dimension of the ranging light beam was n=9, and the farthest range was $d_{Max}=20$ m, while the largest output of angle was $\delta_{\max}=0.3$ rad. The desired $path_{d}$ was the centerline of middle lane. The errors were $\varepsilon_{1}=\varepsilon_{2}=0.1m,\varepsilon_{3}=0.5236$, the continuous action penalty coefficient was $\lambda_{1}=0.01$, and the random initial position was $x_{0}\in \left[10,980\right],y_{0}\in \left[10,50\right]$, $\varphi_{0}\in \left[-\pi /2,\pi /2\right]$. The maximum step number was $Num_{\max}=600$, and the step gap was 0.1 s. The software environment was a Linux operating system with 16 Gb of memory, and the graphics card was a GTX1080 Ti (NVIDIA, Santa Clara, CA, USA). The system took advantage of the deep learning framework TensorFlow.

The greater the learning rate is, the lower the effect of previous training being retained. Similarly, the greater the discount factor is, the more emphasis is placed on experience. The smaller the discount factor is, the more attention is paid to the current return. If the numbers of hidden layers and hidden layer neurons are too little, then the data cannot be fitted well. Conversely, if the numbers of hidden layers and hidden layer neurons are too large, this can easily lead to over-fitting. Therefore, a better network structure and network parameters were designed after several trials. The hyper parameters in the deep reinforcement learning model $M_{\theta }$ were set as follows: the discount factor was γ=0.9, the learning rates of the Actor and Critic networks were both $10^{-4}$, the optimization method of Adam [32] was adopted, the soft update rate was τ=0.001, the number of hidden layer neurons was 512, the size of the experience replay pool was $10^{4}$, the size of the batch was 64, the error was generated by a Gaussian process, the initial variance was $var_{\max}=2$, the minimum variance was $var_{\min}=0.01$, and the attenuation rate was $10^{-4}$.

At low speed, the vehicle dynamic model can be approximated as a bicycle model with two degrees of freedom [4]. The vehicle dynamics model is described in Equation (13).

(13) $$\{\begin{array}{c} \dot{x}=U\cos\varphi -v\sin\varphi \\ \dot{y}=U\sin\varphi +v\cos\varphi \\ \dot{\varphi }=\omega \\ \begin{array}{l} \dot{v}=-\frac{C_{f}+C_{r}}{mU}v-\left(\frac{aC_{f}-bC_{r}}{mU}+U\right)\omega +\frac{C_{f}}{m}\delta \\ \dot{\omega }=\frac{bC_{r}-aC_{f}}{I_{Z}U}v-\frac{a^{2}C_{f}+b^{2}C_{r}}{I_{Z}U}\omega +\frac{aC_{f}}{I_{Z}}\delta \end{array} \end{array},$$

where (*x*, *y*) is the location of the vehicle, φ is the yaw angle, ω is the yaw rate, δ is the front-wheel steering angle, *U* is the longitudinal velocity, and *v* is the lateral velocity. The definitions and values of the vehicle parameters in Equation (13) are shown in Table 1.

The vehicle status is represented by $x={\left[x,\;y,\;\varphi ,\;v,\;\omega \right]}^{T}$, and thus, Equation (13) can be expressed as Equation (14).

(14) $$\dot{x}=f(x,\delta ).$$

**Figure 7:**
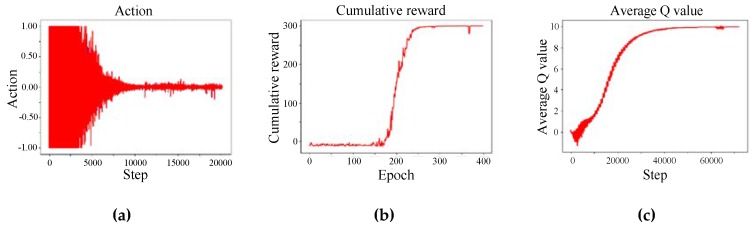

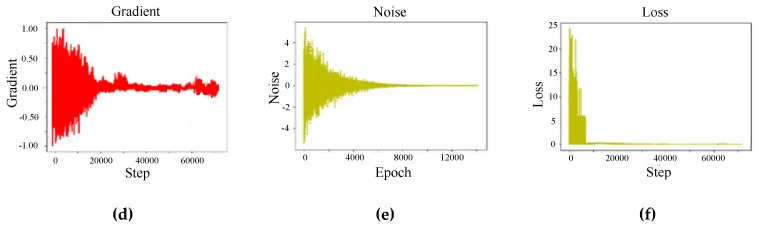
Training results of the deep reinforcement learning model.

Figure 7 shows the changes in parameters during training. Figure 7a has an abscissa of “step” and an ordinate of “action”. Figure 7b has an abscissa of “epoch” and an ordinate of “cumulative reward”. Figure 7c has an abscissa of “step” and an ordinate of “average Q-value”. Figure 7d has an abscissa of “step” and an ordinate of “gradient”. Figure 7e has an abscissa of “epoch” and an ordinate of “noise”. Figure 7f has an abscissa of “step” and an ordinate of “loss”.

As shown in Figure 7, the rewards and the average Q-value of the agent gradually increased with the number of iterations during the training process. Finally, it tended to be stable. On the other hand, the loss gradually decreased to 0 with the increase in the number of iterations, indicating the evaluation of the network becoming more and more effective. The noise decreased as the number of iterations increased, providing sufficient exploration capability in the early stages and sufficient exploitation capability in the late stages. Figure 7 shows that the model learned from experience, and continuously approached the optimal strategy.

### 4.2. Verification of Intelligent Vehicle Trajectory Planning Based on DRL-MTTP

#### 4.2.1. Trajectory Planning of Lane Keeping

**Figure 8:**
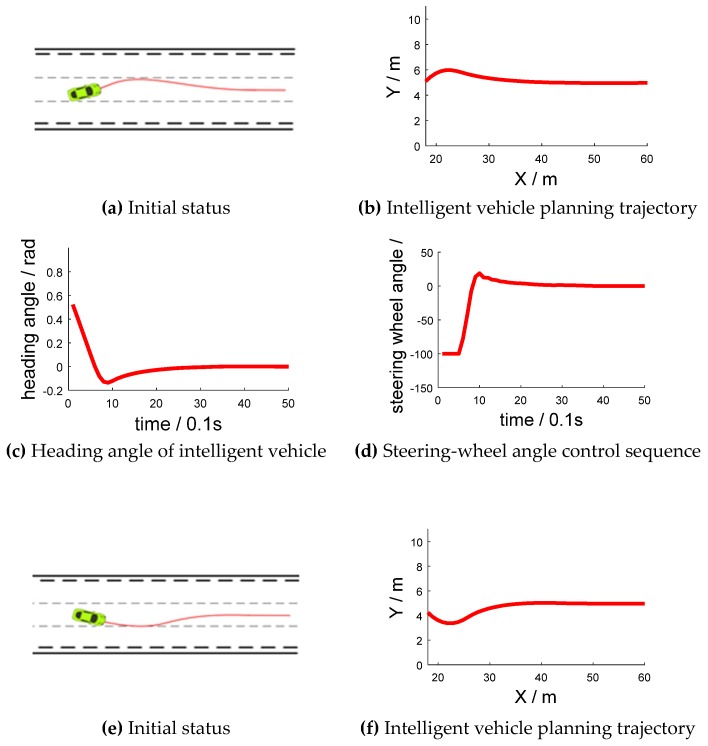

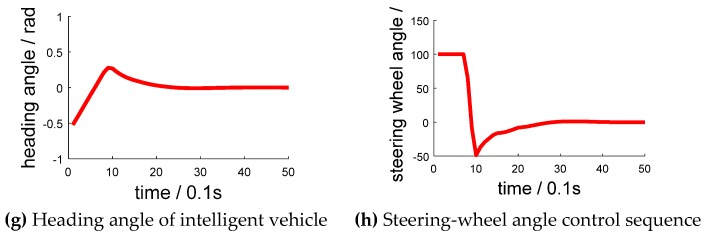
Lane retention experiment.

During the simulation test, the intelligent vehicle speed was set as 10 m/s (36 km/h). The intelligent vehicle started from the center position of the middle lane at a 30° yaw angle and a −30° yaw angle, which are shown in Figure 8a,e, respectively. Figure 8b,f have abscissas of “X/m” and ordinates of “Y/m”. Figure 8c,g have abscissas of “time/0.1 s” and ordinates of “heading angle/rad”. Figure 8d,h have abscissas of “time/0.1 s” and ordinates of “steering-wheel angle/°”.

Through the algorithm model, the steering-wheel control sequence is adjusted to keep in its lane. In the beginning, the intelligent vehicle had a deviation from the initial position, and it then corrected itself to keep to the lane. The average value of front-wheel angle across the three experiments was −0.0001635 rad (−0.009367°). Therefore, the steering angle was stable around 0°, with a mean value of −0.1562°. The average lateral deviation after stabilization was 0.04 cm.

#### 4.2.2. Curve Track Planning

**Figure 9:**
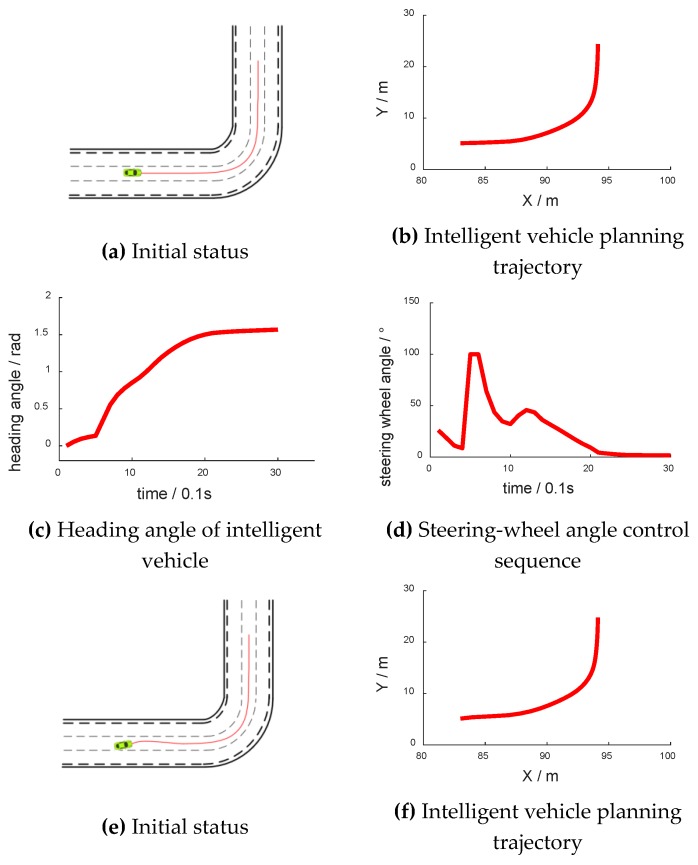

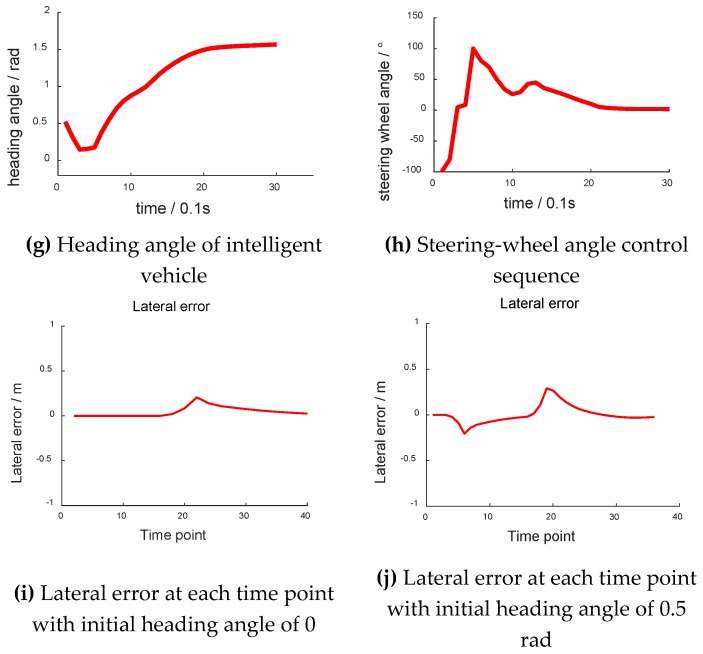
Curve driving experiment.

During the simulation test, the intelligent vehicle set out with different yaw angles, which are shown in Figure 9. Figure 9b,f have abscissas of “X/m” and ordinates of “Y/m”. Figure 9c,g have abscissas of “time/0.1 s” and ordinates of “heading angle/rad”. Figure 9d,h have abscissas of “time/0.1 s” and ordinates of “steering-wheel angle/°”. Figure 9i,j have abscissas of “time point” and ordinates of “lateral error/m”. Figure 9i,j show the lateral error from the center of the lane at each time point. When the vehicle was on the left side of the lane, the lateral error was negative. As shown in Figure 9i, the lateral error was nearly 0 when going straight. When the vehicle turned, the lateral error increased to about 0.2 m. After turning, the lateral error decreased to nearly 0. As shown in Figure 9j, as the initial heading angle was 0.5 rad, and the absolute value of the vehicle lateral error increased first before decreasing to nearly 0. The following trend was the same as that in Figure 9i.

Although there was a shock in the curve, the intelligent vehicle still ultimately navigated the curve successfully. Meanwhile, the curve of the control sequence and the change rate of the yaw angle were relatively smooth.

### 4.3. Experimental Comparison and Analysis of the Three Trajectory Planning Methods

Currently, there are three trajectory-planning methods comparisons and experimental analyses, as described in this section. These trajectory-planning methods are called the optimal trajectory-planning method based on a cubic polynomial [33], the end-to-end trajectory-planning method [9,10], and the model transfer trajectory-planning method based on deep reinforcement learning. The intelligent vehicle drove following the planning results in each simulation step with the same tracking control error. Here, the experiments only compare and analyze the performance of the planning. The traditional trajectory-planning method adopts the angle control sequence based on a preview window. However, the end-to-end trajectory planning method outputs the angle control sequence directly. The period of the trajectory planning was 100 ms. In other words, the intelligent vehicle was reprogrammed after keeping the same deflection angle for 100 ms.

#### 4.3.1. Arc Straight Track Scene

**Figure 10:**
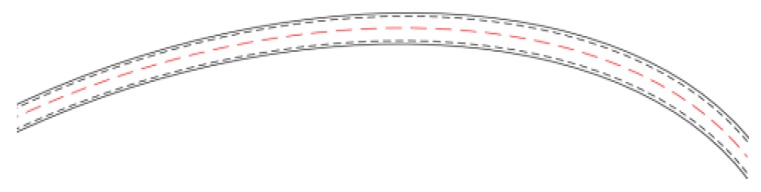
Arc straight track scene diagram.

**Figure 11:**
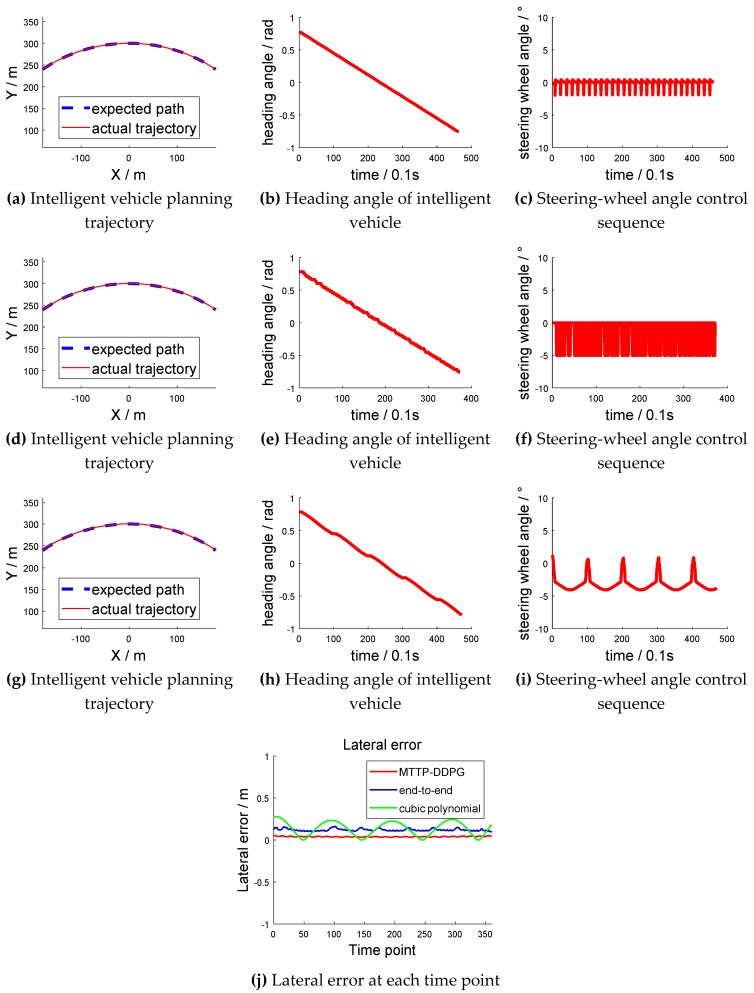
Contrast experiment of arc straight track scene.

The arc shown in Figure 10 had a radius of 300 m; thus, it was large enough to be considered as a straight line in a small range. However, the curvature was not equal to zero. The contrast experiment is shown in Figure 11. Figure 11a–c are the experimental results based on the cubic-polynomial dynamic optimal trajectory-planning method. Figure 11d–f are the experimental results of the end-to-end trajectory-planning method. Figure 11g–i are the experimental results of the model transfer trajectory-planning method based on deep reinforcement learning. Figure 11j shows the lateral error from the center of the lane at each time point. Figure 11a,d,g have abscissas of “X/m” and ordinates of “Y/m”. The blue dotted lines in the figures represent the “expected path” and the red solid lines represent the “actual trajectory”. Figure 11b,e,h have abscissas of “time/0.1 s” and ordinates of “heading angle/rad”. Figure 11c,f,i have abscissas of "time/0.1 s" and ordinates of “steering-wheel angle/°”. Figure 11j has an abscissa of "time point" and an ordinate of "lateral error/m".

The actual trajectories, represented by the solid line, in the three methods were basically the same as the expected trajectory, represented by the dotted line. However, the rotation-angle control sequence of the first two methods oscillated continuously in the small range. The control sequence of our proposed method showed a periodic and continuous variation. The experimental results verified that the model transfer trajectory-planning method based on deep reinforcement learning performs well, and the control action is continuous when driving nearly straight. As shown in Figure 11j, when adopting the optimal trajectory-planning method based on the cubic polynomial, the trajectory of the vehicle was prone to oscillation. There was a medium mean lateral error when adopting the end-to-end trajectory-planning method, while there was a minimum mean lateral error when adopting the method based on MTTP/DDPG.

#### 4.3.2. S-type Ramp Scene

**Figure 12:**
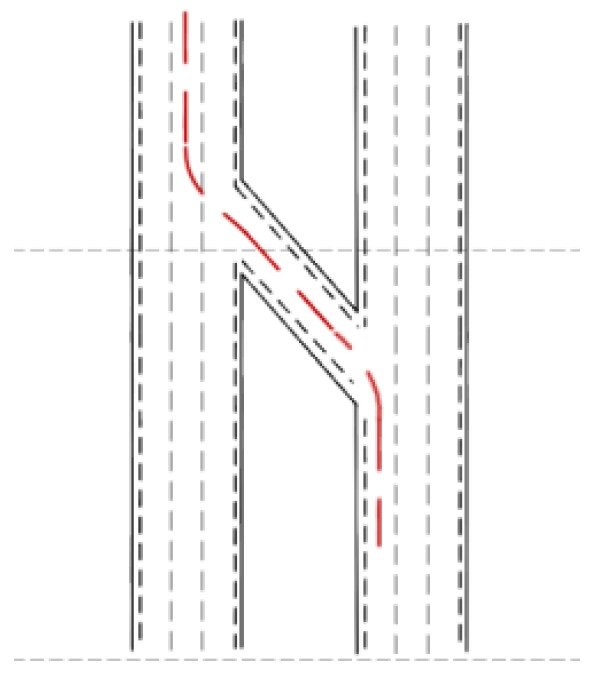
Schematic diagram of the S ramp.

**Figure 13:**
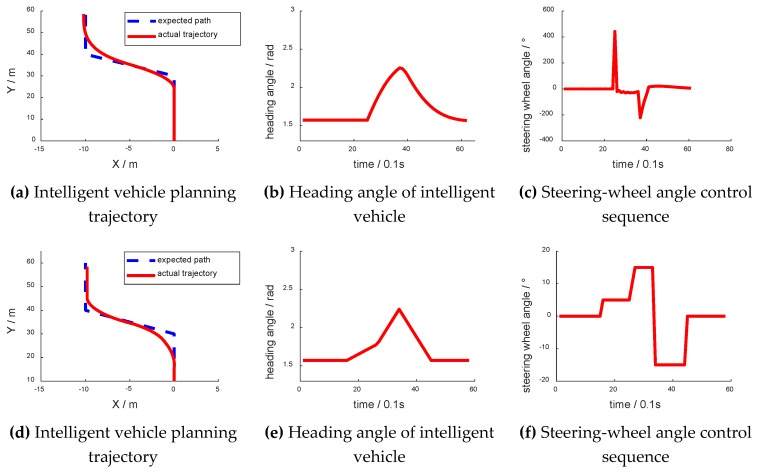

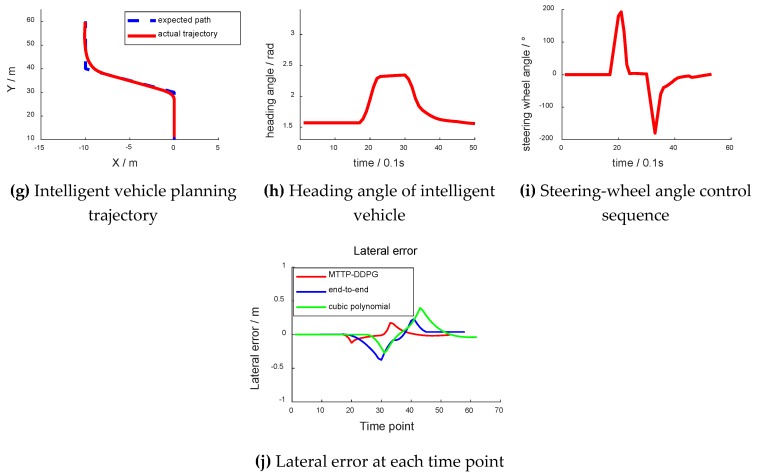
Contrast experiment of S-type ramp scene.

Figure 12 is the schematic diagram of the S ramp. The intelligent vehicle was initially situated in the left lane of the lower right corner. After driving at a yaw angle from the north, the vehicle turned left and entered a 45° ramp. After driving a distance, it turned right in the middle lane and completed the intelligent driving maneuver. The experiment results are shown in Figure 13. Figure 13a–c are the experimental results based on the cubic polynomial dynamic optimal trajectory planning. Figure 13d–f are the experimental results of the end-to-end trajectory planning. Figure 13g–i are the experimental results of the model transfer trajectory-planning method based on deep reinforcement learning. Figure 13j shows the lateral error from the center of the lane at each time point. The abscissas of Figure 13a,d,g are “X/m”, and the ordinates are “Y/m”. The blue dotted line in Figure 13a represents the “expected path” and the red solid line represents the “actual trajectory”. Figure 13b,e,h have abscissas of “time/0.1 s” and ordinates of “heading angle/rad”. The abscissas of Figure 13c,f,i are “time/0.1 s”, and their ordinates are “steering-wheel angle/°”. Figure 13j has an abscissa of "time point" and an ordinate of "lateral error/m".

According to the comparison results between actual trajectory and desired path based on the three planning methods, the results show that the proposed method allowed the intelligent vehicle to maintain a better turning performance, especially in the combination of straight and curved roads. Finally, the lateral deviation of our proposed method was the least, and the it outperformed the other two methods. When entering the ramp or leaving the ramp, there was a greater lateral error. In general, the average lateral error adopting the MTTP/DDPG method was the minimum.

## 5. Real Vehicle Verification of Dynamic Trajectory Planning

The vehicle’s real-time position was collected using the GPS (Global Positioning System) and IMU (Inertial Measurement Unit). The sensory data of the surroundings were acquired using a camera, lidar, and millimeter-wave radar, which were mounted on the driverless vehicle. 

The camera was adopted to detect lane lines. The identification of obstacles depended mainly on the radar and lidar. The fusion process of obstacle information detected by multiple sensors was as follows: firstly, each sensor extracted obstacles accordingly. Then, the Hungarian algorithm (HA) [34] was adopted to match each object extracted by each sensor. Finally, the matched data were fused with the Kalman filter (KF) [35] to get the fusion data.

The lane’s centerline was taken as the desired path based on the lane line detected by the camera. Different points on the desired path were taken as $P_{target}$. After the model transfer strategy, a set of trajectories was obtained. Considering the constraints of the fused obstacle information, the optimal trajectory with no collisions was selected according to the cost function.

**Figure 14:**
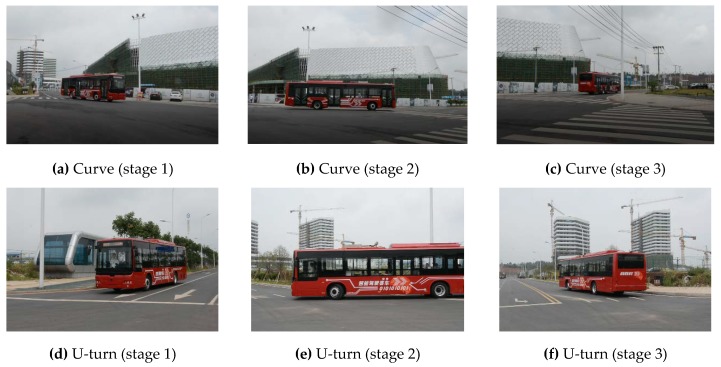

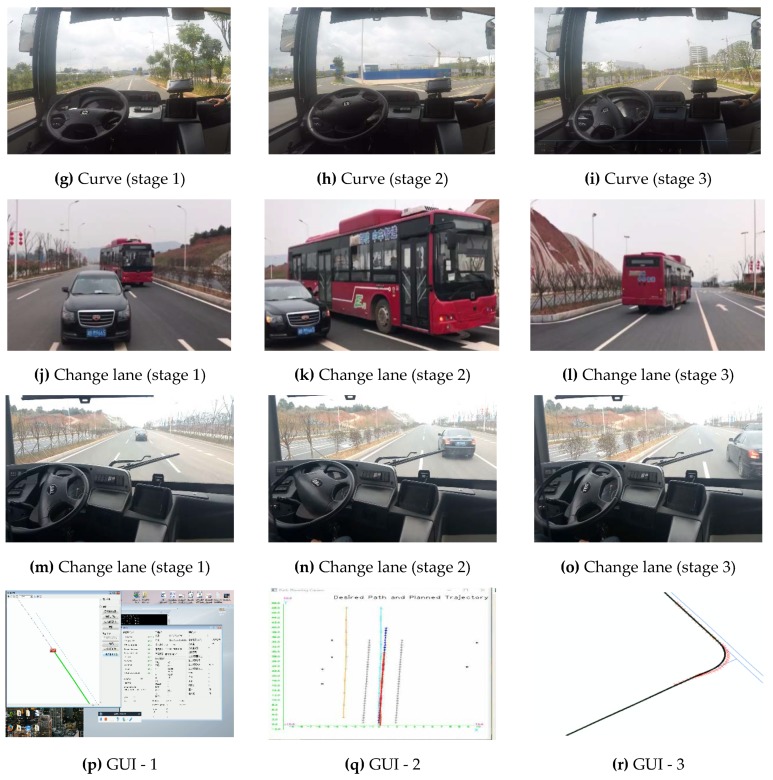
Experimental results of the real vehicle.

Figure 14 shows the actual vehicle’s dynamic trajectory planning process. Figure 14a–i were captured when the vehicle was turning in different scenes. Here, Figure 14a–f are the outside view, while Figure 14g–i are the inside view of the driverless vehicle. Figure 14 shows that the driverless performance of the intelligent vehicle was stable and efficient when turning around 90°. Figure 14j–o were captured when the driverless vehicle was overtaking and lane changing. Figure 14j–l show the outside view and Figure 14m–o show the internal view. They indicate that the intelligent vehicle achieved self-overtaking and lane-changing driving behaviors safely and stably. Figure 14p–r are real-time screenshots and the interface of the intelligent driving trajectory planning. Figure 14r is the tracking result when the actual vehicle verified the curve driving.

## 6. Conclusions

It is difficult for traditional trajectory-planning method to eliminate errors due to vehicle models and road conditions. Furthermore, there are no vehicle dynamics constraints. A model transfer trajectory-planning method based on deep reinforcement learning was proposed in this paper. At first, the complex real environment was abstracted using MTTP, then the abstracted model was transferred into a simple virtual environment through the transfer model. Secondly, the optimal intelligent driving maneuver after deep reinforcement learning training was applied to obtain the optimal control-trajectory sequence in the virtual environment. Thereby, the end-to-end trajectory planning of the intelligent vehicle in a real environment was realized. Moreover, an evaluation function was designed to estimate the planning validity of the control-trajectory sequences, and to judge the risk of collision in a real environment. Furthermore, an optimal control-trajectory sequence was decided and executed by the intelligent land vehicle. Finally, the comparison analysis of multiple driving scenes and multiple trajectory-planning methods verified the better optimization performance of MTTP, showing that it achieved a more continuous rotation-angle control sequence and a smaller lateral error for the intelligent land vehicle. However, the speed of the vehicles was assumed as a constant, and that they were driving in a typical structured environment. Because the virtual simulation environment in this paper did not consider turning around, the proposed method is not applicable when the actual road curvature is very large. The next stage will be to further consider variable-speed driving and more complex environments.

## Figures and Tables

**Table 1:** Vehicle parameters.

Features	Symbols	Parameters
Complete vehicle kerb mass /kg	*m*	17,800
Length	*l*	11,950
Width	*h*	2540
Vehicle yaw moment of inertia /kg·m^2^	$I_{Z}$	20,000
Distance from center of mass to front axle /m	*a*	2.795
Distance from center of mass to rear axle /m	*b*	3.105
Wheel base /m	*d*	5.9
Cornering stiffness of front wheel /N·rad^−1^	$C_{f}$	6500
Cornering stiffness of rear wheel /N·rad^−1^	$C_{r}$	5200

## Nomenclature

VAE	Variational auto-encoder
GAN	Generative adversarial network
end-to-end	Algorithm for inputting original data and outputting result
DQN	Deep Q network
OU	Ornstein–Uhlenbeck
DRL	Deep reinforcement learning
MC	Monte Carlo
TD	Temporal difference
AC	Actor-Critic
DDPG	Deep deterministic policy gradient
MTTP	Model transfer trajectory planning
BN	Batch normalization
HA	Hungarian algorithm
KF	Kalman filter
